# Hydrogel chitosan sorbent application for nutrient removal from soilless plant cultivation wastewater

**DOI:** 10.1007/s11356-018-2078-z

**Published:** 2018-04-26

**Authors:** Tomasz Jóźwiak, Artur Mielcarek, Wojciech Janczukowicz, Joanna Rodziewicz, Joanna Majkowska-Gadomska, Magdalena Chojnowska

**Affiliations:** 1 Department of Environmental Engineering, University of Warmia and Mazury in Olsztyn, ul. Warszawska 117a, 10-957 Olsztyn, Poland; 2 Department of Horticulture, University of Warmia and Mazury in Olsztyn, ul. Prawocheńskiego 21, 10-957 Olsztyn, Poland

**Keywords:** Greenhouse wastewaters, Sorption, Orthophosphates, Nitrates, Chitosan, Hydrogel

## Abstract

In this study, we determined the effectiveness of removal of nutrients (nitrates and orthophosphates) from greenhouse wastewaters (GW) using non-cross-linked chitosan (CHs) and chitosan cross-linked with epichlorohydrin (CHs-ECH) in the form of hydrogel beads. GW used in the study had the following parameters: N-NO_3_ 621.1 mg/L, P-PO_4_ 60.8 mg/L, SO_4_^2−^ 605.0 mg/L, Cl^−^ 0.9 mg/L, Ca^2+^ 545.0 mg/L, Mg^2+^ 178.0 mg/L, K^+^ 482.0 mg/L, hardness 113° dH, and pH 6.2. The scope of the study included determination of the effect of pH on wastewater composition and effectiveness of nutrient sorption, analyses of nutrient sorption kinetics, and analyses of the effect of sorbent dose on percentage removal of nutrients from GW. CHs-ECH was able to sorb 79.4% of P-PO_4_ and 76.7% of N-NO_3_ from GW, whereas CHs to remove 92.8% of P-PO_4_ and 53.2% of N-NO_3_.

**Electronic supplementary material:**

The online version of this article (10.1007/s11356-018-2078-z) contains supplementary material, which is available to authorized users.

## Introduction

Greenhouse wastewaters (GW) are a special type of agricultural wastewaters that are generated in greenhouses during soilless cultivation of vegetables and fruits. They are mainly composed of a fertilizing medium which leached out from the root growth zone and therefore are rich in nitrates and orthophosphates and poor in organic carbon. GW contain also significant amounts of sulfates, potassium, calcium, and magnesium.

Plant fertilization in greenhouses may be conducted in open or closed systems. In the closed system, GW rich in nutrients are collected, supplemented with lacking minerals, and re-used as a fertilizer, while in open systems, they are discharged directly to the ground or—in greenhouses with a concrete floor—to the sewerage system (Dyśko et al. 2013).

In fear of inducing diseases of plants through their contact with wastewater, producers rarely chose the open systems. Today, GW are usually discharged unmanaged to the ground (Saxena and Bassi 2013), and by this means may contribute to the eutrophication of surface waters and adversely affect the quality of underground waters (Prystay and Lo 2001).

GW may pose threat to the natural environment even if discharged to the sewerage system. Considering their untypical composition (high concentrations of N-NO_3_ and P-PO_4_, and low concentration of TOC), their treatment with conventional biological methods based on the activated sludge technology is extremely difficult (Bugajski et al. 2015). Wastewater dilution or feeding the system with an external source of carbon are often cost-ineffective. For this reason, search is underway for alternative treatment methods of wastewaters from soilless plant cultivation in greenhouses.

The most effective methods of phosphorus removal from wastewater include chemical ones like precipitation with calcium and precipitation with salts of iron and aluminum. They are, however, relatively expensive, cause water salinity, and generate high amounts of sludge (Liang et al. 2014). According to the World Health Organization, the best methods for nitrate removal from wastewater are ionic exchange, reversed osmosis, and electrodialysis. Unfortunately, they are very expensive and also generate undesirable by-products (Shrimali and Singh 2001).

A growing interest has recently been observed in the use of sorption as a method for the removal of nutrients from aqueous solutions. Both its costs and effectiveness depend largely on the type of sorbent applied. Sorbents investigated so far regarding their capability for nutrient sorption from aqueous solutions include, e.g., commercial ionic exchangers (Orlando et al. 2002; Samatya et al. 2006; Mustafa et al. 2010), activated carbons (Mishra and Patel 2009; Demiral and Gündüzoǧlu 2010; Zhang et al. 2011), fly ashes (Ragheb 2013), lignocellulosic wastes from the agri-food industry (Orlando et al. 2002; Wang et al. 2007; Huang et al. 2015), and chitosan sorbents (Jóźwiak et al. 2014, 2016, 2017a; Filipkowska et al. 2014) (Table 1).

**Table 1:** Effectiveness of nutrient sorption on different sorbents

Sorbent type	Nutrient type	Q (mg/g)	Reference
Chitosan in the form of hydrogel beads—cross-linked with epichlorohydrin	P-PO_4_	139.40	Filipkowska et al. (2014)
Amberlite IRA-400 (commercial ionic exchanger)	P-PO_4_	121.70	Mustafa et al. (2010)
Biochar from soybean stover	P-PO_4_	76.92	Karunanithi et al. (2017)
Chitosan in the form of hydrogel beads—non-cross-linked	P-PO_4_	44.40	Filipkowska et al. (2014)
Chitosan Zr-biocomposite	P-PO_4_	40.27	Kumar and Viswanathan (2017)
Activated carbon fiber	P-PO_4_	5.85	Zhang et al. (2011)
Kaolinite	P-PO_4_	5.55	Matusik (2014)
Weathered shale	P-PO_4_	3.62	Huang et al. (2015)
Willow wood biochar	P-PO_4_	1.93	Dugdug et al. (2018)
Biochar from cocoa shell	P-PO_4_	1.48	Hale et al. (2013)
Biochar from corn cobs	P-PO_4_	0.17	Hale et al. (2013)
Chitosan in the form of hydrogel beads—cross-linked with epichlorohydrin	N-NO_3_	38.47	Jóźwiak et al. (2014)
PuroliteA 520E (commercial ionic exchanger)	N-NO_3_	35.42	Samatya et al. (2006)
Wheat residue modified with epichlorohydrin	N-NO_3_	29.12	Wang et al. (2007)
Amberlite IRA-900* (commercial ionic exchanger)	N-NO_3_	21.00	Orlando et al. (2002)
Sugarcane bagasse	N-NO_3_	19.60	Orlando et al. (2002)
Rice hull	N-NO_3_	18.20	Orlando et al. (2002)
Commercial activated carbon	N-NO_3_	17.08	Mishra and Patel (2009)
Corn stover biochar	N-NO_3_	8.68	Chintala et al. (2013)
Activated carbon from sugar beet bagasse	N-NO_3_	6.22	Demiral and Gündüzoǧlu (2010)
Eggshell	N-NO_3_	6.07	Ahmad et al. (2017)
Oxidizedcarbon AG-5	N-NO_3_	0.18	Gierak and Łazarska (2017)
Chitosan in the form hydrogel beads—cross-linked with epichlorohydrin	Equimolar mixture (P-PO_4_/N-NO_2_/N-NO_3_)	62.01 (38.22/13.09/10.70)	Jóźwiak et al. (2017a)
Chitosan in the form of hydrogel beads—non-cross-linked	Equimolar mixture (P-PO_4_/N-NO_2_/N-NO_3_)	25.84 (15.72/5.22/4.90)	Jóźwiak et al. (2016)
Fly ashes	Mixture (P-PO_4_/N-NO_3_)	2.76 (2.53/0.23)	Ragheb (2013)

The most efficient sorbents of nitrates and phosphates include chitosan sorbents in the form of hydrogel beads (Table 1). Chitosan is a polysaccharide, a deacetylated form of chitin which is the main constituting material of carapaces of the arthropods and of cell walls of fungi. Chitosan may be acquired on the industrial scale from carapaces of crabs or shrimps—namely from the sea fruit processing industry. Relatively low costs of its acquisition make chitosan an increasingly often used sorbent. Chitosan sorbents proved their high effectiveness also during removal of heavy metals (Kuczajowska-Zadrożna et al. 2016) and dyes from aqueous solutions (Jóźwiak et al. 2013, 2015; Filipkowska and Józwiak 2013).

The high sorption effectiveness of nitrates and phosphates onto chitosan sorbents is due to the presence of amine groups capable of protonation in a polysaccharide structure (−NH_2_ + H^+^ ➔ −NH_3_^+^). The amine groups bearing a positive charge attract electrostatically NO_3_^−^, HPO_4_^2−^, and H_2_PO_4_^−^ anions, which significantly enhances their sorption (Jóźwiak et al. 2016, 2017a). The high effectiveness of nutrient sorption on chitosan beads is also attributed to their hydrogel form which facilitates nutrient access to sorption sites located in deeper layers of the sorbent. The sorption effectiveness of chitosan in the form of hydrogel may be higher by even 224% than that of the same chitosan in the form of flakes (Jóźwiak et al. 2017c). Sorption effectiveness of nitrates and phosphates onto chitosan sorbents may also be increased through chitosan cross-linking with, e.g., epichlorohydrin. It acidifies the interior of hydrogel beads, which results in their swelling, in a greater surface of the sorbent, and also in a stronger interaction with anionic sorbates (Jóźwiak et al. 2017b). The capacity of chitosan cross-linked with epichlorohydrin for the sorption of phosphates may be higher by 214% compared to the non-cross-linked chitosan (Filipkowska et al. 2014) (Table 1).

The use of chitosan sorbents for the removal of nutrients from GW seems to be an ideal method for their treatment and neutralization. Investigations conducted so far have addressed sorption of nutrients from mono- or few-component solutions (nutrient/nutrients + distilled water). In the case of real wastewaters, nutrient sorption may be impaired by the presence of other compounds like, e.g., chlorides or sulfates, which may compete for the active sites of the sorbent with nitrates and phosphates. Presumably, nutrient access to sorption centers may also be hindered by cations of di- or tri-valent metals which may bind with chitosan via complexation (Rashid et al. 2016).

In this study, we examined the effectiveness of removal of nitrates and orthophosphates from GW using non-cross-linked chitosan and chitosan cross-linked with epichlorohydrin in the form of hydrogel beads. The scope of the study included determination of pH effect on the form of nutrients and effectiveness of their sorption, analyses of nutrient sorption kinetics, and analyses of the effect of sorbent dose on the effectiveness of nutrient removal from GW.

## Materials

### Greenhouse wastewaters

GW used in the study originated from soilless cultivation of tomatoes. They were averaged and collected since September 1, 2016 to October 31, 2016 to IBC-type tanks protected against solar radiation. Cultivation area reached 100 m^2^. Coconut fiber served as the substratum for three cultivars of tomatoes: Torero F1, Listell F1, and Growdena F1. A nutrient solution applied to the root system was prepared from tap water, calcium nitrate, saltpeter, potassium monophosphate, potassium sulfate, magnesium sulfate, iron chelate, and a ready-to-use mix of microelements. Doses of the above components were adjusted to the growth phase of plants and weather course. GW used in the study had the following parameters: N-NO_3_ 621.1 mg/L, P-PO_4_ 60.8 mg/L, SO_4_^2−^ 605.0 mg/L, Cl^−^ 0.9 mg/L, Ca^2+^ 545.0 mg/L, Mg^2+^ 178.0 mg /L, K^+^ 482.0 mg/L, hardness 113° dH, and pH 6.2.

### Chitosan

Chitosan (DD = 85%) was obtained from shrimp shells. The manufacturer’s declared viscosity was 500 mPa. Concentration of heavy metals was Pb 20 ppm, Hg 0.2 ppm, and Cd 0.5 ppm. Characteristics of the FTIR spectrum of the chitosan material and sorbents used in experiments were provided in earlier works addressing the use of chitosan sorbents for the removal of nutrients from aqueous solutions prepared under laboratory conditions (Jóźwiak et al. 2016, 2017a).

### Chemical reagents

The following chemical reagents were used in the study: 99% epichlorohydrin (Acros Organics, Poland), 35–38% hydrochloric acid (POCH S.A., Poland), sodium hydroxide in microgranules (POCH S.A., Poland), and 99.5–99.9% acetic acid (POCH S.A., Poland). All reagents were analytically pure or of higher grade.

## Methods

### Preparation of sorbent in the form of hydrogel beads (CHs)

Twenty-five gram d.m. of chitosan in the form of flakes, 925 g of water, and 50 g of acetic acid were added to a 2000-mL beaker. The solution was mixed with a mechanical stirrer and left for 12 h for deaeration. The resulting solution with chitosan concentration of 2.5% was instilled with a syringe into a 2 M solution of sodium hydroxide to form chitosan beads 2.0–2.2 mm in diameter. The formed sorbent was stored in a solution of NaOH for 24 h. Afterwards, the chitosan beads were drained on a screen and rinsed with distilled water to remove residues of sodium hydroxide. Thus, prepared sorbent (CHs) was stored in distilled water in a laboratory cooler (4 °C).

### Preparation of cross-linked chitosan beads (CHs-ECH)

Ten gram d.m. of hydrogel chitosan beads was weighed into a 300-mL conical flask; then, 100 mL of an epichlorohydrin solution with the concentration of 29.0 g/L was added. Concentration of the cross-linking agent was adjusted so as to ensure the 1:1 ratio of functional to amine groups. Next, the solution was placed in a shaker in a water bath (150 r.p.m.). The cross-linking process was conducted at a temperature of 60 °C for 24 h; afterwards, the cross-linked chitosan was rinsed with distilled water to remove residues of epichlorohydrin. The cross-linked chitosan sorbent (CHs-ECH) was stored in distilled water at 4 °C.

### Determination of pH effect on concentrations of nitrate and orthophosphate ions in GW

Eight hundred milliliters of GW was poured to each of 10 beakers (1000 mL). Next, pH value of wastewaters was adjusted in the subsequent beakers to 2.0/3.0/4.0/5.0/6.0/7.0/8.0/9.0/10.0. No pH adjustment was conducted in the last beaker. The adjustment consisted in dosing 1 M solutions of HCl or NaOH into wastewaters. Next, the beakers were placed in a multi-station magnetic stirrer (150 r.p.m.) for 2 h. Afterwards, samples of wastewaters (10 mL) were collected to determine concentrations of nutrients.

### Determination of pH effect on sorption effectiveness of nitrates and orthophosphates

The sorbent (in doses of 1.0 g d.m.) was weighed into a series of conical flasks (500 mL), which were then filled with wastewaters (200 mL) with pH 2–10. Next, the flasks were placed in a laboratory shaker (150 r.p.m.). After 2-h sorption, samples of solutions (10 mL) were collected to determine concentrations of nitrates and orthophosphates remaining in GW.

### Kinetic analyses of the sorption of nitrates and orthophosphates in GW

Five gram d.m. of the chitosan sorbent was weighed into each of two beakers (1000 mL), and 25 g d.m. of the sorbent to another two beakers (1000 mL). Afterwards, wastewaters with optimal sorption pH (determined in point 3.3) were added to two beakers with the weighed sorbent, and wastewaters without pH adjustment were added to another two beakers. Then, the beakers were placed on a magnetic stirrer (150 r.p.m.). Samples (10 mL) were collected from the solutions in specified time intervals (0, 5, 10, 20, 30, 45, 60, 90, 120, 150, 180, 240, 360, 480 min) for the analysis of nitrates and phosphates left in the solutions.

### Determination of the effect of sorbent dose on total composition of GW

Sorbent was weighed into two series of 500-mL conical flasks so as to ensure 0.2/1.0/2.0/5.0/10.0 g d.m. of the sorbent in each series of the subsequent flasks. Then, 200 mL of wastewaters with optimal pH (determined in point 3.4.) was added to each flask of the first series, whereas 100 mL of wastewaters with non-adjusted pH to each flask of the second series. The flasks were placed on a shaker (150 r.p.m.), for the time needed to reach sorption equilibrium determined in point 3.5. Afterwards, samples (10 mL) were collected from the flasks for analyses of concentrations of P-PO_4_, N-NO_3_, SO_4_^2−^, Cl^−^, K^+^, Ca^2+^, and Mg^2+^ left in GW.

### Determination of nutrient concentration

Concentrations of nutrients in wastewaters were determined according to Polish Standards: P-PO_4_—PN-EN 6878:2006—and N-NO_3_—PN-73/C-04576/06. Concentrations of other components (sulfates, chlorides, ions of calcium, magnesium, and potassium) were determined using HACH-type cuvette tests (HACH LANGE Sp. z o.o).

### Computation methods

Quantity of nutrient adsorbed by chitosan was determined from the formula

1 $$ Q=\left(\left({C}_0-{C}_S\right)\times V\right)/m $$

where

*Q*: quantity of sorbed nutrient (mg/g)
*C*_*o*_: nutrient concentration before sorption (mg/L)
*C*_*s*_: nutrient concentration after sorption (mg/L)
*V*: solution volume (L)
*m*: sorbent mass (g d.m.)

Pseudo-first-order (2) and pseudo-second-order (3) equations were used to describe nutrient sorption kinetics:

2 $$ \Delta q/\Delta t={k}_1\times \left({q}_e-q\right) $$

3 $$ \Delta q/\Delta t={k}_2\times {\left( qe-q\right)}^2 $$

where

*k*_*1*_: constant in pseudo-first-order equation (L/min)
*k*_*2*_: constant in pseudo-second-order equation (L/min)
*q*_*e*_: equilibrium quantity of sorbed nutrient (mg/g)
*q*: momentary quantity of sorbed nutrient (mg/g)

A simplified model of intramolecular diffusion (4) was used to describe nutrient sorption stages:

4 $$ {q}_t={k}_d\times {t}^{0.5} $$

*q*_*t*_: momentary quantity of sorbed nutrient (mg/g d.m.)
*k*_*d*_: intramolecular rate constant (mg/g min^−0.5^)
*t*: sorption time (min)

### Laboratory equipment

The following laboratory equipment was used in the study: HI 221 pH meter, Hanna Instruments (USA)—pH measurement and adjustment, Genesys 20 spectrophotometer, Thermo Scientific (USA)—nutrient concentration assay, MS-53M Magnetic Stirrers, GMI (USA)—nutrient sorption (kinetics), SK-71 Shaker, Jeio Tech (South Korea)—nutrient sorption, JWE-357 water bath with shaking, JW Eelectronic (Poland)—chitosan cross-linking.

## Results and discussion

### Effect of pH on concentrations of nitrates and orthophosphates in GW

Apart from nitrates (621.1 mg N-NO_3_/L) and orthophosphates (60.8 mg P-PO_4_/L), the GW contained also other components like sulfates (605.0 mg SO_4_^2−^/L) as well as ions of calcium (545.0 mg Ca^2+^/L) and magnesium (178.0 mg Mg^2+^/L), which were likely to affect the sorption process. Considering a negative charge of their ions, sulfates were expected to compete with nitrates and orthophosphates for the sorption centers of chitosan, thus reducing their sorption effectiveness. In turn, depending on pH, divalent metals Ca^2+^/Mg^2+^ could precipitate orthophosphates and thus affect their concentration in wastewaters. For this reason, analyses of pH effect on the sorption effectiveness of nutrients on chitosan sorbents were preceded by investigations of pH effect on the concentration of nutrients in the solution.

**Fig. 1:**
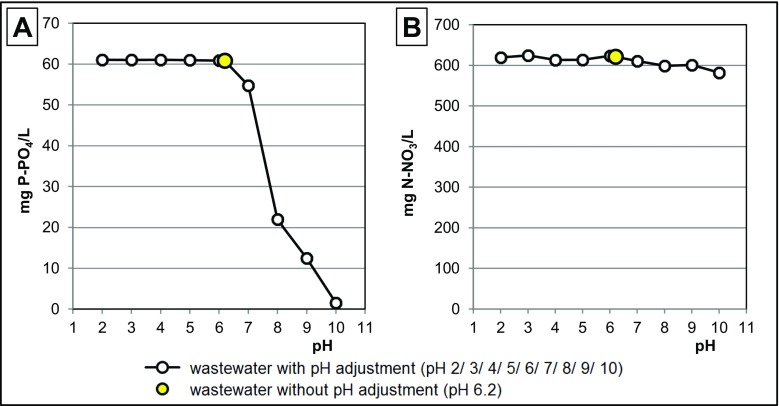
Effect of pH on concentrations of **a** P-PO_4_ and **b** N-NO_3_ in GW. Temp. 22 °C

In a pH range of 2.0–6.2, i.e., below the natural pH value of GW, the concentration of orthophosphates remained at a similar level. Above pH 6.2, the concentration of P-PO_4_ in wastewaters began to successively decrease and reached 64.0 and 2.5% of the initial value at pH 8.0 and pH 10.0, respectively (Fig. 1a). This effect was due to the precipitation of orthophosphates with ions of calcium and magnesium, the efficiency of which increased along with pH increase. At acidic pHs, orthophosphates remained in the non-dissociated form. Experimental results suggest that in the case of GW rich in Ca^2+^/Mg^2+^ ions, the concentration of orthophosphates may be modified by pH adjustment.

In turn, pH adjustment had no significant effect on the concentration of nitrates in GW (Fig. 1b). It resulted from the fact that salts of nitrates are usually very easily soluble and do not precipitate even at high pH values.

### Effect of pH on nutrient sorption effectiveness

**Fig. 2:**
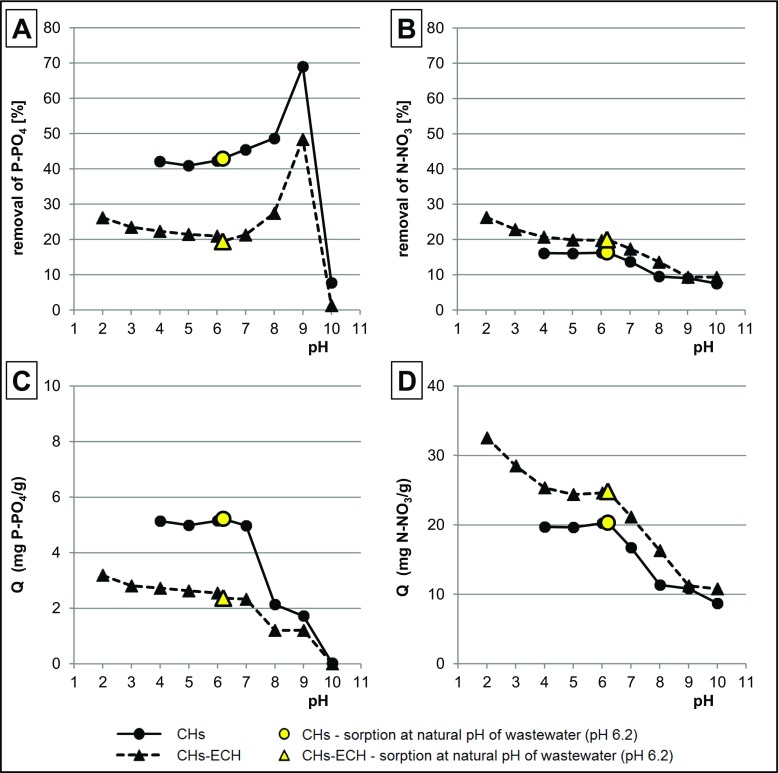
Effect of pH on the effectiveness of **a** P-PO_4_ and **b** N-NO_3_ removal from GW and quantity of sorbed **c** P-PO_4_ and **d** N-NO_3_ on tested sorbents. Temp. 22 °C

During sorption with the use of CHs and CHs-ECH, at the initial pH range of pH 5–9, the effectiveness (%) of P-PO_4_ removal from GW increased along with pH increase (Fig. 2a). However, it does not have to indicate increased affinity of orthophosphates to sorbents along with pH increase. As it results from point 4.1 (Fig. 1a), with pH increase from 6.2 up, P-PO_4_ concentration in wastewaters decreased due to precipitation of orthophosphates with calcium and magnesium cations. Due to the low initial P-PO_4_ concentration in wastewaters, caused by their initial precipitation (pH < 6.2), the percentage removal of phosphates onto CHs and CHs-ECH was relatively high. Already at pH > 9, the affinity of phosphates to sorbents was low enough to cause marginal effectiveness of their sorption even at very low initial concentration of P-PO_4_ (Fig. 2a).

The effect of pH on the real quantity of P-PO_4_ removed using CHs and CHs-ECH is presented in Fig. 2c. In the case of CHs, at initial pH 4–7, the mass of P-PO_4_ bound with the sorbent was similar; however, the best result was achieved at pH 4.0 and 6.2 (without pH adjustment). The non-cross-linked chitosan solvent dissolved at pH < 4. For this reason, results of nutrient sorption onto CHs at pH 2–3 were not depicted in Fig. 2. At pH > 7, sorption effectiveness was observed to significantly decrease. The lowest quantity of orthophosphates sorbed onto CHs was determined at pH 10.

The quantity of P-PO_4_ removed onto CHs-ECH was the highest at pH 2 and decreased along with pH increase in the entire analyzed pH range to reach the lowest value at pH 10. The decrease in the effectiveness of orthophosphate sorption onto CHs-ECH was not uniform in the tested pH range (Fig. 2c), with the greatest decreases noted at pH 2–3 and pH 7–10.

Considering that the pH value had no greater effect on the content of dissociated nitrate ions in GW (Fig. 1b), removal of N-NO_3_ depended mainly on the quantity of nitrates sorbed on the sorbents (Fig. 2b, d). At initial pH 4.0–6.2, the effectiveness of N-NO_3_ sorption onto CHs was similar; however, the best results were achieved at pH 4.0 and pH 6.2, like in the case of P-PO_4_. At pH > 6.2, the effectiveness of N-NO_3_ sorption onto CHs decreased with pH increase and reached the lowest value at pH 10. Likewise for P-PO_4_, the effectiveness of N-NO_3_ sorption onto CHs-ECH decreased along with pH increase in the whole tested range of pH values, with the best result achieved at pH 2 and the worst at pH 10. A similar tendency was observed in studies on the use of chitosan for the sorption of nutrients from aqueous solutions (nutrient + distilled water) (Jóźwiak et al. 2014; Filipkowska et al. 2014).

The increased sorption effectiveness of nutrients onto chitosan sorbents along with decreasing initial pH value of the sorption process results from the presence of amine groups in a chitosan structure. The primary amine groups are capable of simple protonation: −NH_2_ + H_3_O^+^ → −NH_3_^+^ + H_2_O. Positively charged amine groups attract electrostatically anions present in the solution, which significantly enhances their sorption (Piccin et al. 2009). The higher the number of protonated amine groups, the lower the solution pH. With pH increase, the number of protonated amine groups decreases, which has a negative impact on sorption effectiveness of anions. At high pH values, sorbent surface may gain a negative charge, owing to which it electrostatically repulses particles with a negative charge; this in turn inhibits their sorption (Szymczyk et al. 2016). In the alkaline environment, the sorption effectiveness of anions may be additionally reduced by their competition with OH^−^ ions for the sorption centers of chitosan. This explains the low effectiveness of sorption of nitrates and orthophosphates on chitosan sorbents at pH > 7.

CHs displayed much higher capability for P-PO_4_ sorption compared to CHs-ECH. A completely different effect was reported in the study wherein sorption was conducted on solutions of phosphates in distilled water (Filipkowska et al. 2014). The effectiveness of P-PO_4_ sorption onto CHs-ECH, obtained in that study, was incomparably higher than on CHs. These differences in phosphate sorption effectiveness between wastewaters and solutions of nutrients based on distilled water are due to, i.e., the presence of calcium and magnesium ions in wastewaters. This will be discussed in detail in point 4.3.

**Fig. 3:**
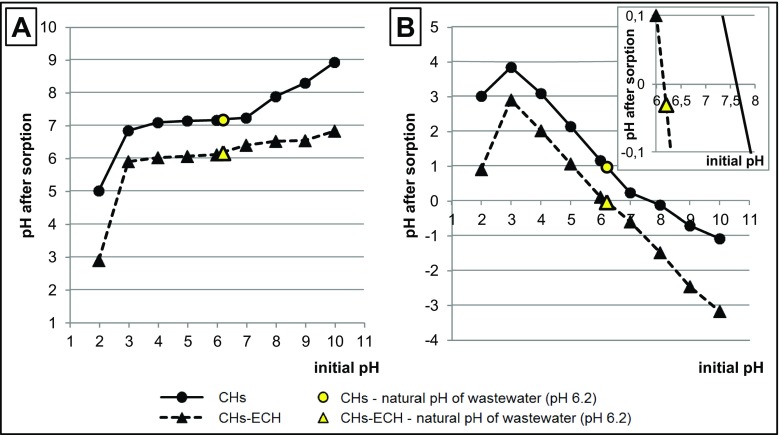
**a** Effect of sorbent on change in GW pH. **b** Point pH_PZC_ of tested sorbents

The analyzed chitosan sorbents affected wastewater pH after the sorption process (Fig. 3a). During sorption with the use of CHs, at initial pH 3–7, the final pH value of wastewater was between pH 6.8 and pH 7.8. In the case of CHs-ECH, at initial pH 3–10, the pH value of GW after sorption ranged from pH 5.9 to pH 6.8. Changes in wastewater pH during sorption result from solution tending to point pH_PZC_ (point of zero charge (PZC)), being typical of the tested sorbent (Jóźwiak et al. 2017b). The pH_PZC_ values determined for the analyzed sorbents (Fig. 3b) reached pH_PZC_ = 7.6 for CHs and pH_PZC_ = 6.2 for CHs-ECH.

### Effect of sorption on total composition of GW

The objective of this study was to determine the extent of removal of major components of wastewaters from soilless cultivation of tomatoes in greenhouses during sorption with the use of CHs and ECH-CHs (Table 2). Apart from nitrates and orthophosphates, special attention was paid to sulfates and also to calcium and magnesium ions. Potentially, the analyzed wastewater components could affect the sorption of P-PO_4_ and N-NO_3_. The experiment was conducted for two variants of pH (natural pH of wastewater, without adjustment, and optimal pH determined in point 4.2.) and for two doses of the sorbent (5 and 50 g/L) (Table 2).

**Table 2:** Percentage removal of major components of GW during sorption onto CHs and CHs-ECH

Component	Crude wastewater (mg/L)	Removal of wastewater components during sorption (%)							
		Sorbent dose 5 g/L				Sorbent dose 50 g/L			
		CHs	CHs	CHs-ECH	CHs-ECH	CHs	CHs	CHs-ECH	CHs-ECH
		Without adjustment pH 6.2	pH 4	Without adjustment pH 6.2	pH 2	Without adjustment pH 6.2	pH 4	Without adjustment pH 6.2	pH 2
P-PO_4_ (mg/L)	60.80	46.9	43.4	21.2	27.8	92.8	92.6	75.7	79.4
N-NO_3_ (mg/L)	621.1	17.6	16.8	21.0	27.3	53.2	49.0	71.8	76.6
SO_4_^2−^ (mg/L)	605.0	10.7	10.7	48.8	72.1	38.8	38.1	86.8	91.7
Cl^−^ (mg/L)	0.9	–	–	–	–	–	–	–	–
K^+^ (mg/L)	482.0	6.6	13.7	15.8	18.7	14.7	26.1	16.6	23.9
Ca^2+^ (mg/L)	545.0	13.6	11.7	6.1	4.2	51.8	51.8	34.5	32.3
Mg^2+^ (mg/L)	178.0	21.9	20.2	16.9	12.9	42.7	43.5	18.0	17.4
Hardness (^0^dH)	113.0	13.0	11.5	6.2	3.5	46.5	46.9	32.3	6.2

Besides nitrates and orthophosphates, crude GW had a high concentration of sulfates (605.0 mg SO_4_^2−^/L), which could compete with nutrients for the sorption centers of chitosan. Sulfate ions were especially effectively bound onto CHs-ECH. Already at CHs-ECH dose of 5 g/L, the effectiveness sulfate removal reached 48.8% at the natural pH of wastewaters (pH 6.2) and 72.1% at their pH 2. Considering good solubility of calcium and magnesium sulfates, it was assumed that SO_4_^2−^ ions were not removed from the system through their precipitation.

The higher sulfate sorption capability of CHs-ECH than of CHs resulted most probably from differences in the structure of sorbents. Due to HCl synthesis during cross-linking, the pH value inside a hydrogel bead of CHs-ECH was low. Excess H_3_O^+^ ions present in the hydrogel-induced protonation of amine functional groups of chitosan, which led to electrostatic repulsion between polysaccharide chains and, ultimately, to sorbent swelling. Breakdown of the hydrogel sorbent was, however, impossible due to solid covalent cross-linking. The swollen structure of CHs-ECH resulted in greater availability of sorption centers (Jóźwiak et al. 2017b). The higher number of protonated amine groups resulted also in stronger interactions with anions present in wastewaters. The more effective sorption of anions onto CHs-ECH than on CHs was also observed in the case of nitrates (Table 2). The higher sorption capability of CHs-ECH compared to CHs was also shown in studies on the sorption of nitrates and orthophosphates and of “mono-component” dyes from solutions based on distilled water (distilled water + sorbate) (Jóźwiak et al. 2014; Filipkowska et al. 2014).

The achieved effectiveness of P-PO_4_ sorption from GW onto CHs was much higher than onto CHs-ECH. Most likely, this was due to the presence of divalent ions of metals in the solution (Ca^2+^/Mg^2+^). After pH increase to the value close to pH_PZC_ for CHs (pH_PZC_ = 7.6), part of orthophosphates present in the solution were, presumably, precipitated in a wastewater solution or on the surface of CHs. In the case of CHs-ECH, due to low wastewater pH (pH < 6.2), the precipitation of orthophosphates with Ca^2+^ and Mg^2+^ ions was little likely. For this reason, the quantity of orthophosphates removed from GW during sorption onto CHs was higher than during sorption onto CHs-ECH. As CHs-ECH showed significantly lower effectiveness of SO_4_^2−^ sorption compared to CHs, the lower effectiveness of P-PO_4_ removal onto CHs-ECH could also result from their competition with sulfates for the remaining active sites.

The removal of calcium and magnesium ions during sorption with the use of CHs-ECH resulted mainly from their complexation on sorbent’s surface. Considering a low number of free hydroxyl groups, the quantity of Ca^2+^ and Mg^2+^ ions sorbed onto CHs-ECH was, however, relatively small. Quantities of removed calcium and magnesium were higher during sorption onto CHs, but—besides sorption/complexation—a significant part of Ca^2+^ and Mg^2+^ ions was removed from the system as a result of precipitation with orthophosphates.

The tested chitosan sorbents removed also potassium ions from GW. At sorbent dose of 5 g/L and natural pH of GW, the effectiveness of K^+^ sorption reached 6.6% onto CHs and 15.8% onto CHs-ECH. Presumably, the mechanism of potassium removal from wastewater consisted in its interaction with anions sorbed on the surface of the chitosan sorbent. The larger surface of CHs-ECH explained the higher sorption effectiveness of K^+^ ions.

### Kinetics of nutrient sorption onto CHs and CHs-ECH

Analyses of the course of sorption process in time were conducted at the optimal pH, adjusted for individual sorbents and at natural pH of GW (without pH adjustment).

**Fig. 4:**
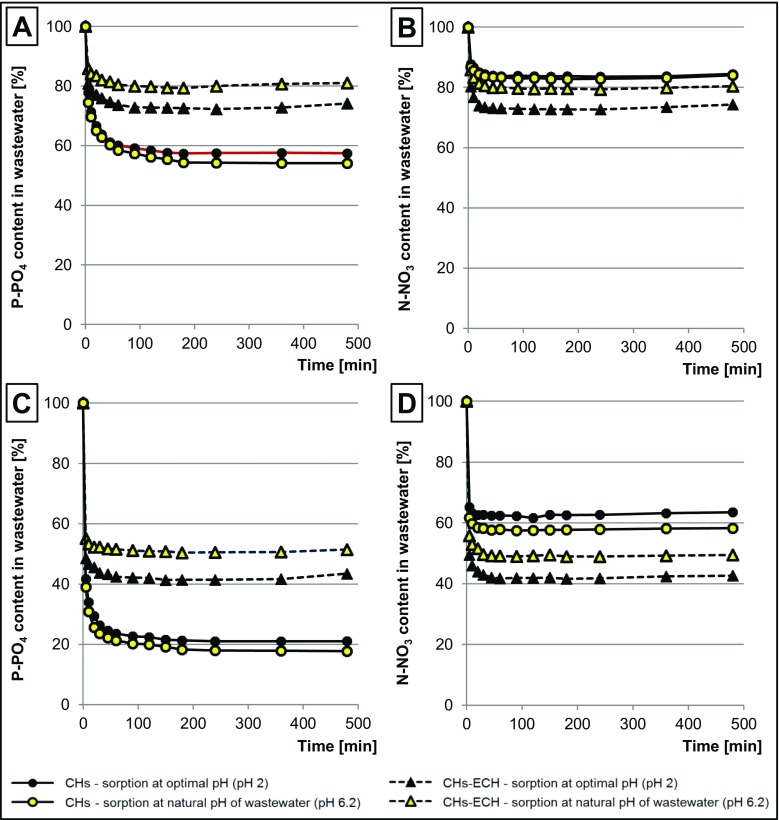
Changes in concentrations of nutrients in GW during sorption. **a** P-PO_4_, dose of sorbents—5 g/L. **b** N-NO_3_, dose of sorbents—5 g/L. **c** P-PO_4_, dose of sorbents—25 g/L. **d** N-NO_3_, dose of sorbents—25 g/L. Temp. 22 °C

Regardless of sorbent dose and sorption process pH, the equilibrium time of P-PO_4_ sorption onto CHs reached 180 min (Fig. 4a, b). In the case of sorption of orthophosphates onto CHs-ECH, it was difficult to determine sorption equilibrium in the analyzed time interval because after 240 min of the process, part of P-PO_4_ bound on the sorbent began to desorb. In turn, time after which the quantity of P-PO_4_ sorbed onto CHs-ECH was the highest reached 180 min, irrespective of pH value and sorbent dose.

In studies on chitosan sorbent use for the sorption of nutrients from an equimolar mixture of P-PO_4_, N-NO_2_, and N-NO_3_ (nutrients + distilled water), the time needed to reach the maximum sorption effectiveness of P-PO_4_ ranged from 60 to 120 min (Jóźwiak et al. 2016). The shorter sorption time of orthophosphates from solutions based on distilled water was, probably, due to the absence of other substances which would compete for the active sites of chitosan (calcium/magnesium ions, sulfates).

The highest intensity of P-PO_4_ sorption onto chitosan sorbents was observed at the first stage of the process. The rate of orthophosphate sorption was also significantly affected by sorbent dose. During wastewater treatment with CHs, already after 10 min, the quantity of P-PO_4_ removed at CH dose of 5 g/L reached 66–67% and at CH dose of 25 g/L—83–84% of the value determined in the equilibrium state (Fig. 4a, c). In the case of CHs-ECH, the quantity of phosphates sorbed within the first 10 min reached 76–78 and 91–04% of the maximum value (*Q*_max_) at sorbent doses of 5 and 25 g/L, respectively.

As in the case of P-PO_4_ sorption onto CHs-ECH, the time needed to reach the equilibrium of N-NO_3_ sorption onto chitosan sorbent was difficult to establish owing to the phenomenon of nitrate desorption. Time after which the quantity of N-NO_3_ bound with the sorbent was the highest depended on sorption pH and ranged from 45 to 60 min for both CHs and CHs-ECH (Fig. 4b, d). In studies conducted with aqueous solutions of nutrients (distilled water + nutrients), time after which a similar quantity of N-NO_3_ was bound with chitosan was alike and fitted in the range from 30 to 60 min (Jóźwiak et al. 2016).

Likewise in the case of P-PO_4_, the effectiveness of N-NO_3_ sorption onto chitosan sorbents was the highest at the initial stage of the sorption process. Upon the use of CHs, already after 10 min of the process, the quantity of N-NO_3_ bound on the sorbent reached 81–84 and 95–98% of *Q*_max_ value at sorbent doses of 5 and 25 g/L, respectively. During sorption onto CHs-ECH, within the first 10 min, the mass of sorbed N-NO_3_ reached 81–85% of *Q*_max_ at sorbent dose of 5 g/L and 92–93% of *Q*_max_ at sorbent dose of 25 g/L.

The phenomenon of nutrient desorption after a specified time of the sorption process was also observed in investigations with the use of chitosan sorbent for the removal of a mixture of nutrients from aqueous solutions based on distilled water (Jóźwiak et al. 2016, 2017a) The established cause of partial desorption of nutrients was a change in wastewater pH induced by solution tending to reach pH_PZC_ of the sorbent. In the case of GW, desorption of P-PO_4_ from CHs was, probably, impaired by precipitation of phosphates with ions of calcium and magnesium present in the top layers of the hydrogel structure of the sorbent.

Experimental data of nutrient sorption from GW in time were described with the pseudo-first and pseudo-second-order models (Table 3). Values of the determination coefficient (*R*^2^) indicate that the kinetics of sorption of nitrates and phosphates on each tested sorbent was best described by the pseudo-second-order model. In the case of both CHs and CHs-ECH, the determined reaction rate constant *K*_2_ depended on sorbent dose and was higher at a higher dose of chitosan. The *K*_2_ value was also affected, though to a lesser extent, by pH value of the solution the process of sorption was conducted in. Higher values of the reaction rate constant were achieved at natural pH of GW (pH 6.2).

**Table 3:** Kinetic parameters of nutrient sorption onto CHs and CHs-ECH determined from the pseudo-first and pseudo-second-order model. Temp. 22 °C

Nutrient	Sorbent dose	Sorbent type	Sorption pH	Pseudo-first-order model			Pseudo-second-order model			Exp. data
				*K*_1_	*q*_*e*_	*R* ^2^	*K*_2_	*q*_*e*_	*R* ^2^	*q*_*e*_, (exp)
				(1/min)	(mg/g)	–	(g/mg min)	(mg/g)	–	(mg/g)
P-PO_4_	5 (g/L)	CHs	4	0.1233	5.09	0.9693	0.0354	5.44	0.9983	5.34
			6.2^a^	0.1362	5.30	0.9456	0.0368	5.67	0.9901	5.71
		CHs-ECH	2	0.2244	3.28	0.9602	0.1142	3.44	0.9912	3.42
			6.2^a^	0.2185	2.41	0.9527	0.1477	2.53	0.9874	2.56
	25 (g/L)	CHs	4	0.2598	1.91	0.9843	0.2684	1.98	0.9991	1.98
			6.2^a^	0.2692	1.97	0.9871	0.2792	2.04	0.9993	2.06
		CHs-ECH	2	0.4388	1.43	0.9915	0.9015	1.46	0.9981	1.46
			6.2^a^	0.5006	1.21	0.9970	1.5980	1.22	0.9994	1.23
N-NO_3_	5 (g/L)	CHs	4	0.2514	19.15	0.9820	0.0261	19.83	0.9977	19.65
			6.2^a^	0.2824	20.08	0.9824	0.0291	20.74	0.9979	20.69
		CHs-ECH	2	0.2413	32.23	0.9946	0.0155	33.30	0.9985	32.69
			6.2^a^	0.2177	24.08	0.9890	0.0169	25.04	0.9996	24.63
	25 (g/L)	CHs	4	0.5198	9.20	0.9990	0.1732	9.28	0.9991	9.19
			6.2^a^	0.4680	10.22	0.9981	0.2753	10.35	0.9998	10.28
		CHs-ECH	2	0.4012	14.04	0.9960	0.0861	14.29	0.9998	14.24
			6.2^a^	0.3995	12.10	0.9948	0.0976	12.32	0.9993	12.27

^a^Natural pH of GW

In the case of P-PO_4_ sorption onto CHs, the *q*_*e*_ values calculated based on the pseudo-second-order model indicate that the removal of orthophosphates at natural pH of wastewaters (pH 6.2) and at pH 4 proceeded with a comparable intensity. A similar observation was made for N-NO_3_ sorption onto CHs. The negligibly lower effectiveness of nutrient sorption onto CHs at pH 4 could be due to a lower concentration of chlorides which, presumably, compete with phosphates for the sorption centers of chitosan.

The sorption of orthophosphates onto CHs-ECH was more effective at pH 2 than at pH 6.2. This results from a high number of protonated amine groups at pH 2 which attracted electrostatically the orthophosphate anions. In addition, the low pH facilitated sorbent swelling, thereby increasing permeability of the hydrogel membrane and increasing the accessibility to sorption centers present in the deeper layers of the sorbent (Jóźwiak et al. 2017a, b).

Despite greater accessibility to sorption centers and stronger interaction with amine groups of CHs-ECH, the removal of orthophosphates from GW was more intensive in the case of CHs. Most likely, this was due to precipitation of P-PO_4_ with calcium and magnesium ions in the system with CHs, which was described in detail in point 4.3.

The model of intramolecular diffusion fitted to experimental data (Table 4) indicates that sorption of nutrients onto chitosan proceeded in three stages. At the first stage of sorption, wastewater nutrients were bound in the upper layer of the structure of hydrogel beads, therefore presumably the key role was ascribed to the process of surface adsorption of nutrients. When most of the sorption centers on the hydrogel membrane had been saturated, the sorption process entered into the second stage which consisted mainly in the absorption of nitrates and orthophosphates. At this stage, nutrients began to migrate into the interior of the hydrogel structure and occupy active sites located in deeper layers of the sorbent. The high density of nutrients in the top layers of the sorbent and ionic interactions between nutrients and sorption centers impaired penetration of nutrients into hydrogel’s interior. For this reason, the second stage of the sorption process was longer and less effective than the first one. At the third stage, all available active centers of the sorbent were completely saturated and the sorption reaction reached its equilibrium. This stage of sorption was the longest and the least intensive.

**Table 4:** Rate constants of diffusion of nitrates and orthophosphates determined from the intramolecular diffusion model. Temp. 22 °C

Nutrient	Sorbent dose (g/L)	Sorbent type	Sorption pH	First stage of sorption			Second stage of sorption			Third stage of sorption		
				*k*_d1_	Duration	*R* ^2^	*k*_d2_	Duration	*R* ^2^	*k*_d2_	Duration	*R* ^2^
				(mg g^−1^ min^−0.5^)	(min)	–	(mg g^−1^ min^−0.5^)	(min)	–	(mg g^−1^ min^−0.5^)	(min)	–
P-PO_4_	5	CHs	4	6.5870	5	0.9999	1.1611	25	0.9744	0.0535	120	0.8515
			6.2^a^	7.1613	5	0.9999	0.9653	25	0.9945	0.1272	120	0.6864
		CHs-ECH	2	9.1096	10	0.9776	0.4339	35	0.9081	0.0783	135	0.9239
			6.2^a^	6.6043	10	0.9802	0.7450	35	0.9838	0.0833	135	0.7019
	25	CHs	4	3.8192	5	0.9999	0.2734	15	0.8642	0.0389	100	0.8227
			6.2^a^	4.1595	5	0.9999	0.3525	15	0.9585	0.0293	100	0.6807
		CHs-ECH	2	5.5120	5	0.9999	0.2714	40	0.9705	0.0163	80	0.9128
			6.2^a^	4.7498	5	0.9999	0.2730	40	0.9387	0.0218	80	0.9850
N-NO_3_	5	CHs	4	6.5870	5	0.9999	1.1611	25	0.9744	0.0535	120	0.8515
			6.2^a^	7.1613	5	0.9999	0.9653	25	0.9945	0.1272	120	0.6864
		CHs-ECH	2	9.1096	10	0.9776	0.4339	35	0.9081	0.0783	135	0.9239
			6.2^a^	6.6043	10	0.9802	0.7450	35	0.9838	0.0833	135	0.7019
	25	CHs	4	3.8192	5	0.9999	0.2734	15	0.8642	0.0389	100	0.8227
			6.2^a^	4.1595	5	0.9999	0.3525	15	0.9585	0.0293	100	0.6807
		CHs-ECH	2	5.5120	5	0.9999	0.2714	40	0.9705	0.0163	80	0.9128
			6.2^a^	4.7498	5	0.9999	0.2730	40	0.9387	0.0218	80	0.9850

^a^Natural pH of GW

Regardless of the initial pH of GW, the first stage of orthophosphate removal onto CHs (5 g/L) lasted ca. 10 min. The fivefold increase of CH dose (25 g/L) shortened it to 5 min. At this dose of CHs (25 g/L), shortened was also stage 2 (Table 4).

Already at sorbent dose of 5 g/L, the first stage of P-PO_4_ sorption onto CHs-ECH was very short, which resulted from high affinity of orthophosphates to sorption centers of CHs-ECH [16]. As in the case of CHs, stage 2 shortened as a result of the higher dose of CHs-ECH (25 g/L) (Table 4). Both at sorbent dose of 5 and 25 g/L, stage 1 of sorption of nitrates onto CHs was relatively short and lasted 5 min. Compared to P-PO_4_, shorter was also the time of stage 2 of P-PO_4_ sorption onto CHs, which could result from a lower mass of nitrates compared to orthophosphates. The total duration of stages 1 and 2 of N-NO_3_ sorption onto CHs-ECH was longer than onto CHs. The longer sorption of nitrates onto CHs-ECH was, most likely, due to accumulation of sulfates in the top layers of the hydrogel which impaired penetration of nitrates into the deeper layers of the sorbent.

Intensity of the first two stages of nutrient sorption onto CHs-ECH was largely dependent on the initial pH of wastewater and was higher at pH 2 than at the natural pH of wastewater (pH 6.2). The cause of the greater affinity of nutrients to sorbent at the lower pH value was explained in point 4.3.

Stage 3 of P-PO_4_ sorption onto CHs-ECH ended 60 min before desorption stage began. In the case of N-NO_3_, this period was longer and reached ca. 120 min. A similar interval between stage 3 of sorption and desorption stage was achieved for N-NO_3_ removal onto CHs.

In each case, the effectiveness of nutrient sorption (expressed per gram of dry matter of sorbent) decreased along with sorbent dose increase. This is a typical phenomenon attributed to the competition between sorbents for sorbates available in the solution.

### Effect of sorbent dose on the effectiveness of nutrient sorption from GW

**Fig. 5:**
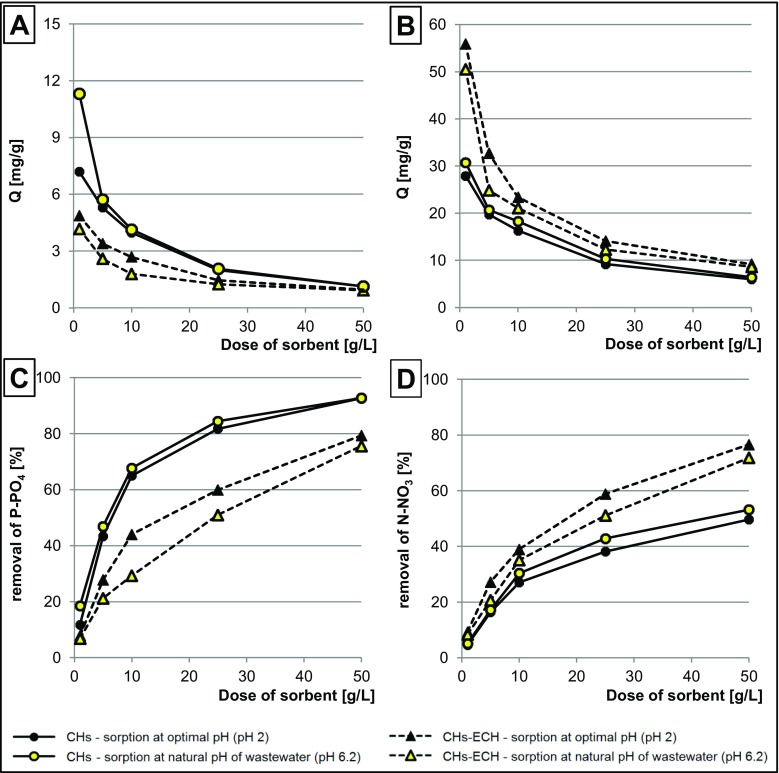
Effect of sorbent dose on the quantity of sorbed nutrients **a** P-PO_4_ and **b** N-NO_3_ and effect of sorbent dose on the removal of nutrients from GW: **c** P-PO_4_ and **d** N-NO_3_. Temp. 22 °C

Sorbent dose had a significant effect on the effectiveness of nutrient sorption from GW. When expressed per gram d.m., sorption effectiveness was increasing along with a decreasing dose of the sorbent. In all experimental series, the highest sorbent saturation with nutrients was determined at sorbent dose of 1 g d.m./L (Fig. 5a, b).

The removal of orthophosphates was the most effective onto CHs, which in a solution of wastewater with natural pH (pH 6.2) at a dose of 1 g d.m./L sorbed as much as 11.3 mg P-PO_4_/g. Under the same conditions, the maximum quantity of phosphates removed onto CHs-ECH reached only ca. 5 mg/g (Fig. 5a, b).

The removal of nitrates from wastewaters was the most effective using chitosan cross-linked with epichlorohydrin. The best nitrate sorption capability was noted for CHs-ECH at pH 2, when 55.9 mg N-NO_3_/g was sorbed at its dose of 1 g d.m./L. For comparison, CHs in the same dose was able to sorb 30.7 mg N-NO_3_/g.

The effect of sorbent dose in particular solutions on the removal of nutrients from GW was depicted in Fig. 5c, d. At pH 2, the 50 g/L dose of CHs-ECH ensured the removal of 77% of N-NO_3_ and 79% of P-PO_4_ from GW (at initial concentration of N-NO_3_ and P-PO_4_ at 621.1 and 60.8 g/L, respectively). Negligibly lower effectiveness of nutrient removal onto CHs-ECH was achieved in the variant without pH adjustment (pH 6.2) (Fig. 5c, d). The fivefold lower dose of sorbent (10 g CHs-ECH/L) removed 39% of nitrates and 44% of orthophosphates from GW.

CHs applied in a dose of 50 g/L (pH 6.2) were able to remove 93% of P-PO_4_ and 53% of N-NO_3_ from GW. An interesting result was obtained already at CH dose of 10 g/L (CHs removed 68% of P-PO_4_ and 27% of N-NO_3_) (Fig. 5c, d).

## Conclusions

- Sorption with the use of appropriately prepared chitosan sorbents may be an effective method for the removal of nitrates and orthophosphates from greenhouse wastewaters.
- High sorption effectiveness may be achieved on condition of using a respectively high dose of the sorbent.
- The components of greenhouse wastewaters which have the greatest impact on the impaired sorption of nitrates and orthophosphates on chitosan sorbent are, probably, sulfate anions as well as cations of calcium and magnesium.
- Chitosan sorbents are capable of changing pH of wastewater, which results from system tending to reach pH value close to pH_PZC_ of sorbent (pH_PZC_ for CHs = 7.6/pH_PZC_ for CHs-ECH = 6.2). In the case of sorption with CHs, the increase in wastewater pH to pH > 7 offered conditions for precipitation of part of P-PO_4_ with Ca^2+^ and Mg^2+^ ions. During sorption with CHs-ECH, the process of P-PO_4_ precipitation was little likely due to a low pH_PZC_ value of the sorbent and low pH of wastewaters (pH ≤ 6.2). The effectiveness of N-NO_3_ sorption from greenhouse wastewaters onto CHs-ECH was higher than on CHs. This is due to greater permeability of the hydrogel membrane of CHs-ECH compared to CHs and also due to higher availability of sorption centers located in the deeper layers of the hydrogel.
- Sorption of N-NO_3_ and P-PO_4_ on the tested chitosan sorbents proceeded in three stages. At the first stage, the most intensive and the shortest one, nutrients occupy sorption centers on the top layers of the hydrogel sorbent. At the second, less intensive but longer stage, sorbates bind with the active sites in deeper layers of the hydrogel. At the third stage, being the longest but the least effective stage, sorption centers are completely saturated.
- The control of sorption time (duration) is significant in processes of greenhouse wastewater treatment using chitosan sorbents. In the case of CHs and CHs-ECH, the contact time of sorbent with wastewaters should not exceed 240 min. Due to high concentrations of calcium and magnesium ions in greenhouse wastewaters, orthophosphates may be precipitated from the system at appropriate pH.
- Theoretically, after sorption of nitrates and orthophosphates from wastewaters, the CHs and CHs-ECH could be used in farming as fertilizers. Apart from nutrient release to the substratum, chitosan sorbents could serve as a hydrogel (substitute for polyacrylane/polyacrylamide) to increase water retention and improve soil structure.

## Electronic supplementary material

ESM 1 (DOCX 354 kb)
