# Supplementary material for: Hydrogel chitosan sorbent application for nutrient removal from soilless plant cultivation wastewater
Source: Environ Sci Pollut Res Int. 2018 Apr 26;25(19):18484–97. doi: 10.1007/s11356-018-2078-z (PMC6061506; doi:10.1007/s11356-018-2078-z)
Supplement: Supplementary file 1 — (DOCX 354 kb) [file 11356_2018_2078_MOESM1_ESM.docx]

Supplement A. Kinetics of nutrient sorption from GW onto CHs and CHs-ECH: A) P-PO_4_, dose of sorbents – 5 g/L, B) N-NO_3_, dose of sorbents – 5 g/L, C) P-PO_4_, dose of sorbents – 25 g/L, D) N-NO_3_, dose of sorbents – 25 g/L. Temp. 22 ^o^C.

Supplement B. Intracellular diffusion model of nutrient sorption: A) P-PO_4_, dose of sorbents – 5 g/L, B) N-NO_3_, dose of sorbents – 5 g/L, C) P-PO_4_, dose of sorbents – 25 g/L, D) N-NO_3_, dose of sorbents – 25 g/L. Temp. 22 ^o^C.
